# Intracranial Pressure Monitoring and Management in Aneurysmal Subarachnoid Hemorrhage

**DOI:** 10.1007/s12028-023-01752-y

**Published:** 2023-06-06

**Authors:** Alberto Addis, Marta Baggiani, Giuseppe Citerio

**Affiliations:** 1 School of Medicine and Surgery, University of Milano-Bicocca, Monza, Italy; 2 Neurological Intensive Care Unit, Fondazione Istituto di Ricovero e Cura a Carattere Scientifico San Gerardo dei Tintori, Monza, Italy; 3 University Eastern Piedmont, Novara, Italy

**Keywords:** Intracranial pressure, Subarachnoid hemorrhage, Review

## Abstract

Aneurysmal subarachnoid hemorrhage is a medical condition that can lead to intracranial hypertension, negatively impacting patients’ outcomes. This review article explores the underlying pathophysiology that causes increased intracranial pressure (ICP) during hospitalization. Hydrocephalus, brain swelling, and intracranial hematoma could produce an ICP rise. Although cerebrospinal fluid withdrawal via an external ventricular drain is commonly used, ICP monitoring is not always consistently practiced. Indications for ICP monitoring include neurological deterioration, hydrocephalus, brain swelling, intracranial masses, and the need for cerebrospinal fluid drainage. This review emphasizes the importance of ICP monitoring and presents findings from the Synapse-ICU study, which supports a correlation between ICP monitoring and treatment with better patient outcomes. The review also discusses various therapeutic strategies for managing increased ICP and identifies potential areas for future research.

## Introduction

Aneurysmal subarachnoid hemorrhage (SAH) remains a devastating condition, with a reported mortality of up to 36% and an elevated chance of poor long-term functional outcomes in survivors [1, 2]. The amount of initial bleeding and developing secondary brain injury through various mechanisms, including early brain injury [3], vasospasm, delayed ischemia, and high intracranial pressure (ICP) [4], significantly affect the patient’s outcome [2].

In recent years, efforts have been focused on methods for the early identification of such intracranial pathophysiological derangements and the development of optimized management strategies to minimize secondary injury. Currently, guidelines for managing high ICP (HICP) in SAH stem from studies in traumatic brain injury (TBI) [5–7]. However, because of the complex and unique pathophysiological milieu of SAH, more focused research is being conducted to identify the peculiarities of ICP management in patients with SAH [8].

## Pathophysiology of Elevated ICP

The most common cause of HICP is hydrocephalus, reported in 30% of patients [2], either communicating or obstructive, followed by intracerebral hemorrhage (ICH) and the development of global cerebral edema (GCE), either early (8%) or delayed (10–12%) [9, 10]. Less common causes are subdural hematoma (5%), cerebral infarction (2–3%), and extracranial causes.

According to pathophysiology, elevated ICP in SAH can present acutely (within 24 h), subacutely (up to 7–10 days), or in a delayed manner. Its development independently predicts poor functional outcomes and increased mortality [4].

The initial rise is linked to aneurysmal bleeding. The sudden release of blood in the subarachnoid space during hemorrhage onset can lead to a rapid increase in ICP, potentially reaching the mean arterial pressure, causing a transient cerebral circulatory arrest, and resulting in loss of consciousness [11]. As such, loss of consciousness at onset is considered a surrogate for cerebral hypoperfusion and is associated with an increased risk of developing early cytotoxic GCE [12] and, consequently, a worse prognosis. Possible additional contributors to early GCE are vasomotor paralysis and a rise in cerebral blood volume. The reported incidence of early GCE varies across studies, between 8 and 67% of patients [9]. Furthermore, early ICP rise can be caused by obstructive or communicating acute hydrocephalus [2, 13]. Most poor-grade or comatose patients will eventually develop hydrocephalus due to cerebrospinal fluid (CSF) blockage.

After 3–4 days post-SAH, the ICP starts normalizing, possibly because of decreasing subarachnoid blood levels, improved CSF dynamics, or reduced brain edema [14]. Prolonged HICP in this context has been independently correlated with the amount of blood on computed tomography scans, rebleeding, early ischemic lesions, and poor neurological status [15]. Subacute or early persistent cerebral edema contributing to HICP seems to be related to the degree of early ischemic injury and cerebral hypoperfusion, with subsequent ion channel dysfunction and resultant cellular swelling [16].

Later in the course of the disease, HICP has been associated with the persistence of hydrocephalus, the mass effect caused by ICH or edema, either cytotoxic (caused by delayed cerebral ischemia aggravated by cerebral metabolic crisis, cortical spreading depolarizations, and seizures) or vasogenic (due to various degree of blood–brain barrier disruption, neuroinflammation, oxidative stress, or altered cerebral autoregulation with cerebral hyperemia).

Hydrocephalus could be communicating, due to impaired CSF absorption by arachnoid granulations, or noncommunicating (obstructive), due to direct blockage of ventricular efflux, typically in cases with an intraventricular extension of the hemorrhage [17]. Early hydrocephalus develops in one fifth to one third of patients after SAH and is more commonly seen in patients with diffuse SAH and those with a poor clinical grade, regardless of aneurysm treatment modality.

Several studies have documented impaired cerebral autoregulation in SAH, mainly in comatose patients [18]. Considering the need to maintain adequate cerebral blood flow during the period at risk for delayed cerebral ischemia through blood pressure augmentation, impaired autoregulation could result in increased ICP. Delayed hydrocephalus can develop in the absence of acute hydrocephalus weeks to months later. Impairment in CSF flow may persist owing to abnormal secretion and reabsorption of the CSF, obstruction of the arachnoid granulations, and adhesions within the ventricular system, [19] requiring permanent CSF diversion in almost a quarter of patients [20].

## Monitoring

### Indications

ICP monitoring practice in patients with SAH is still debated, with no specific existing guidelines for this pathology. The Neurocritical Care Society strongly recommends ICP monitoring in case of acute brain injury at risk of elevated ICP based on clinical and/or imaging features [21]. Moreover, the Neurocritical Care Society consensus on neuromonitoring defined some general indications for ICP monitoring in nontraumatic conditions (i.e., coma, computed tomography findings suggestive of HICP, and neurological worsening) [22]. Unfortunately, no specific indication has yet been tailored to patients with SAH [7]. Generally, indications for ICP monitoring in SAH include a Glasgow Coma Score ≤ 8 or neurological worsening, acute hydrocephalus, the development of cerebral edema (either early or delayed), intracranial masses, and the need for perioperative monitoring or CSF drainage [22, 23].

The insertion of an intraventricular catheter in acute symptomatic hydrocephalus after SAH is also recommended by American Heart Association (AHA)/American Stroke Association (ASA) guidelines (class I, level of evidence B) [5] without suggesting continuous ICP monitoring. Measurement of ICP during CSF drainage with an external ventricular drain (EVD) typically requires stopping the flow during measurement [23]. Therefore, the simultaneous presence of an intraparenchymal transducer, zeroed at the tragus level, allows continuous ICP monitoring without interrupting CSF diversion.

### Ventricular Catheters and Intraparenchymal Devices

The measurement of ventricular fluid pressure using a ventriculostomy connected to a pressure transducer is the current gold standard for measuring ICP, as it is highly accurate, cost-effective, and reliable for monitoring ICP. This method allows for periodic recalibration and therapeutic drainage of CSF, providing clinicians with a valuable tool for managing patients with intracranial hypertension. The intraparenchymal transducer is the second most common device used for ICP monitoring and is also considered reliable in clinical practice. These methods provide clinicians with essential tools to monitor and manage patients with ICP abnormalities [24].

The lack of possibility for CSF drainage limits the use of isolated intraparenchymal probes in aneurysmal SAH. Because ICP monitoring through an open EVD is unreliable and hence relies on periodic closing [25], some centers prefer placing both devices routinely, thus allowing continuous CSF drainage and simultaneous monitoring [26]. Moreover, this approach proves helpful when there are technical limitations to intraventricular monitoring, such as a collapsed ventricle around the catheter compromising the accuracy of measurements [27].

The Synapse-ICU study [28], a prospective observational cohort study that enrolled 423 patients with SAH at 146 intensive care units in 42 countries [29], described the practice of ICP monitoring across centers. ICP was monitored in 295 patients with SAH (69.7%). A ventriculostomy was inserted in 54% of them. Significant between-country variability in ICP insertion was observed, ranging from 4.7 to 79.9% (median odds ratio 3.04). The median duration of ICP monitoring was 12 days (range 8–18), which was longer compared with that in patients with TBI, with a daily median ICP value of 14 mm Hg (interquartile range 10–19 mm Hg) and a median maximum value of 21 mm Hg (interquartile range 16–30 mm Hg).

### Timing and Side of the Insertion

When an EVD is placed before aneurysm occlusion, which has been documented as generally safe [26], it can protect against sudden rises in ICP if rebleeding occurs. However, careful avoidance of large acute CSF withdrawals before the aneurysm is secured is warranted because this has been associated with a higher incidence of rebleeding [30–32]. EVDs are also indicated when there is a need for intrathecal delivery of a thrombolytic agent to help reduce intraventricular hemorrhage, though with unclear outcome benefits [33].

The side of EVD placement also requires evaluation; the ventricle with less blood is usually selected to minimize the risk of clogging the device. However, the compartmentalization of ICP must be considered because ICP is often more significant on the side of maximum pathology.

### Noninvasive Methods

Alternative noninvasive methods for ICP estimation are being pursued [24]. Ultrasound evaluation of optic nerve sheath diameter (ONSD) is often used for triage decisions [34]. ONSD evaluation is possible because the meninges enclose the optic nerve inside the orbit, and the space between them and the nerve directly connects with the intracranial subarachnoid space. Hence, any increase in ICP would transmit along the nerve, thus causing optic nerve sheath dilation. However, probably because of the initial bleeding that stresses the sheath, this technique is not reliable in patients with SAH [35]. Transcranial Doppler, frequently used for monitoring changes in blood flow velocities after SAH, is an ultrasound-based technique that might be useful for a noninvasive assessment of ICP. However, its clinical utility is questionable because of its poor precision [36].

### Threshold for Treatment

An ICP treatment threshold is not clearly defined. Stemming from the trauma literature, a “classical” threshold is around an ICP of 20 mm Hg. Interestingly, the association with poor outcomes occurred at lower ICP values than usually considered [37–39]. Although this might be partially explained by the continuously open EVD resulting in lower ICPs or that ICP elevation is a surrogate marker of underlying mechanisms leading to neurological deterioration, it could also reflect the need to lower our ICP target in SAH further. Of note, Cagnazzo et al. [37] found that ICP significantly influenced the occurrence of delayed cerebral ischemia (DCI)-related cerebral infarction, indicating values of ICP < 6.7 mm Hg as protective against cerebral ischemia; they suggested that a lower ICP threshold in patients with SAH might reduce the pressure around capillary vessels, improving nutritive exchanges with brain parenchyma. These findings are consistent with those of Fugate et al. [38], who found that CSF drainage at low levels of ICP (5 mm Hg) might improve microcirculation and tissue perfusion. Samuelsson et al. [40] studied the relationship between ICP variables and brain tissue metabolism through cerebral microdialysis and found that ICP ≤ 10 mm Hg was associated with a favorable brain tissue metabolism profile.

### The ICP Dose

Recently, some consideration has been given to the fact that absolute ICP values might not be the best tool to guide management of SAH. Alternatively, more advanced methods have been proposed rather than referring to just a threshold value, as it was demonstrated that the concept of “dose,” reflecting both the length and the magnitude of exposure to HICP, better quantifies the ICP burden. This concept was first introduced in the TBI population by Vik et al. [41]. In a multicenter study including 350 patients with SAH, Citerio et al. demonstrated that ICP > 25 mm Hg for more than 5 min is an independent factor associated with unfavorable outcomes [42]. The Monza group [43] showed how exposure to a moderate pressure–time dose of HICP at 20 and 30 mm Hg correlated with 6-month mortality. More recently, Carra et al. [44] investigated the association between ICP dose and long-term neurological outcomes of patients with SAH. The combination of intensity and duration defined the tolerance to intracranial hypertension, and the pressure–time burden correlated with long-term neurological outcomes more closely than the time spent over a fixed threshold of 20 mm Hg, confirming the importance of the ICP dose in patients with SAH.

Because cerebral perfusion pressure (CPP) is the difference between mean arterial pressure and ICP, ICP monitoring is a prerequisite for patients’ CPP-based management. Current evidence more strongly supports CPP-directed care than ICP-directed care [19]. A recent SAH consensus recommended maintaining a CPP of 70 mm Hg, with the arterial transducer zeroed at the level of the tragus for an accurate estimation [23]. Moreover, Ryttlefors et al. [45] found that a CPP > 100 mm Hg was associated with better clinical results.

## Management of Increased ICP in SAH

Specific guidelines for treating raised ICP in patients with SAH are missing, and the current recommendations are extrapolated from the TBI population [5, 46]. The current management strategies for treating raised ICP within the SAH population emphasize critical differences from the TBI population and highlight potential future research directions on this controversial topic [7].

In the Synapse-ICU study [28, 29], episodes of high ICP that required treatment were recorded in 54.7% of patients, occurring less frequently in patients with good neurological status. ICP spikes were more prevalent in patients with a parenchymal device (67.5%) than in those with an intraventricular device (46.1%). Patients who received ICP monitoring also received more aggressive therapy treatments, as indicated by their higher therapy intensity level (TIL) (TIL score 10.33 [standard deviation 3.61]) compared with nonmonitored patients (TIL score 6.3 [standard deviation 4.19], *p* < 0.001). In more severe cases, ICP monitoring and associated treatment were significantly associated with better 6-month outcomes, with lower odds of poor neurological outcome (odds ratio 0.14, 95% confidence interval 0.02–0.53, *p* = 0.0113) and mortality (hazard ratio 0.25, 95% confidence interval 0.13–0.49, *p* < 0.0001). ICP management should ideally be directed to the underlying pathophysiological mechanism to maximize results, and its refractoriness to therapy correlates with clinical gravity [47].

### CSF Withdrawal

CSF withdrawal is a crucial strategy for controlling ICP. The current guidelines for the management of SAH [5] state that SAH-associated acute symptomatic hydrocephalus should be managed by CSF diversion (class I; level of evidence B). It is unclear whether the recommendation of a specific method of CSF drainage (i.e., continuous or intermittent) offers any clinical benefit, and there is a lack of consensus suggesting an optimal approach [22]. Intermittent drainage is “on demand”; the EVD remains closed, and ICP is continuously recorded. The EVD is opened when the patient becomes symptomatic or when ICP increases over a threshold (usually around 15–20 mm Hg for more than 5 min), and it is closed again when a normal ICP value is obtained. In continuous EVD drainage, the drain is left open against a defined water column, usually with the pressure gradient set to 10–15 mm Hg. The clinician could also define the drained volume, usually around 10 mL/hour.

Comparing continuous vs. intermittent EVD approaches, no significant differences have been reported concerning the incidence of vasospasm, ventriculoperitoneal shunt dependency, and length of hospital stay [48]. A recent randomized controlled trial compared continuous CSF drainage to intermittent drainage in 60 patients following SAH. The overall rate of complications was higher in the continuous drainage group, and there were no significant differences in ICP control, rate of DCI, or functional outcome [49]. Data suggest intermittent drainage with rapid weaning may be beneficial with lower rates of permanent shunts, shorter hospital length of stay, and fewer EVD-related complications [50].

EVD weaning and discontinuation are frequently discussed and require further scientific exploration to reach a definitive recommendation. The guidelines for the management of SAH state that the weaning of an EVD over 24 h does not appear to be effective in reducing the need for ventricular shunting (class III; level of evidence B) and that cases of SAH-associated chronic symptomatic hydrocephalus should be treated with permanent CSF diversion (class I; level of evidence C) [5]. Independent predictors of permanent shunting are age, low Glasgow Coma Scale score, elevated CSF protein levels, presence of intraventricular hemorrhage, CSF erythrocyte count, ventriculitis, and duration of EVD. However, despite similar rates of shunt dependency between rapid and gradual weaning, the gradual approach might prolong the length of stay in both the neurointensive care unit and the hospital. A more recent consensus of the Neurocritical Care Society released in 2018 encouraged an EVD to wean as soon as clinically feasible without high-level evidence [51]. Despite these few indications, a recent multi-institutional American survey found that a continuously open EVD to enhance CSF drainage and a gradual EVD wean are preferred to intermittent drainage coupled with rapid weaning [52]. The same result was found in a Scandinavian survey that found poor adherence to national guidelines with EVD discontinuation dependent mainly on patient clinical condition and drainage volume [53].

Of note, routine fenestration of the lamina terminalis, which has previously been suggested to reduce the incidence of shunt-dependent chronic hydrocephalus, seems to add no further clinical benefit and thus should not be routinely performed (class III; level of evidence B).

The effect of thrombolytic administration through EVD has been studied regarding the outcome and rate of shunt dependency in the clot lysis: evaluating accelerated resolution of intraventricular haemorrhage (CLEAR III) trial [33] in the setting of ICH. In a primary analysis comparing irrigation with alteplase versus normal saline, the alteplase group had reduced mortality (odds ratio 0.5, 95% confidence interval 0.31–0.8), although most of the survivors ended up with a severe disability; thus, no difference in reasonable outcome rates was identified. Furthermore, no differences in shunt dependency incidence were found in the intraventricular alteplase group vs. the saline group. Further studies are needed to elucidate any potential benefit of intraventricular thrombolysis, as according to current evidence, it is not recommended as an intervention to improve functional outcomes. However, the possibility of benefit has been hypothesized if greater and faster clot removal could be achieved.

Lumbar drainage is sometimes used as an alternative to external ventricular drainage for patients with SAH, but its use should be carefully considered because of potential risks and uncertain benefits [54, 55]. This approach is intended as a less invasive alternative to prolonged use of external ventricular drainage and relies on restoring normal CSF circulation after clearance of intraventricular clots. Specifically, lumbar drainage should only be considered once obstructive hydrocephalus has been successfully relieved and once CSF outflow from the lateral ventricles via the third and fourth ventricle into the subarachnoid space is no longer impaired. However, the benefits of lumbar drainage for patients with SAH are not clear yet. Although some studies suggest it can improve outcomes, a large high-quality prospective trial failed to show a significant difference in neurological outcomes at 6 months [56]. Additionally, concerns about the safety of lumbar drains remain, and there is no consensus on how they should be incorporated into existing treatment algorithms.

Considering these uncertainties, it is important to approach the usage of lumbar drains in patients with SAH cautiously. Clinicians should carefully weigh the potential benefits against the risks and ensure that patients are closely monitored for adverse effects. No definitive recommendation on lumbar drains in patients with SAH can be made, and further research is needed to understand their potential benefits and risks better.

Further medical management of ICP crises in SAH is extrapolated from algorithms for patients with TBI [57] applying increasingly aggressive interventions.

### Head of the Bed Elevation

In managing raised ICP, elevating the head from 0 to 30 degrees effectively reduces ICP, likely because of the hydrostatic displacement of CSF and facilitated venous outflow from the brain [47].

### Hyperventilation and Hypocapnia

The partial pressure of carbon dioxide (PaCO_2_) is a strong vasomodulator, with a reduction in CO_2_ causing vasoconstriction of cerebral arteries, resulting in reduced cerebral blood volume and hence ICP [58, 59].

This vasoconstrictive effect is only transient, as it lasts only hours. Therefore, hyperventilation is indicated only as a temporary measure to control ICP and should be terminated once the indication ceases. This strategy, even if the effect of local acidosis counteracts cerebral blood flow reduction [60], needs to be used cautiously in the SAH setting, in which maintaining adequate blood flow in the early stages of the disease is a priority.

### Osmotherapy

Osmotherapy is frequently used in patients with SAH. The optimal osmotherapy agent in SAH is debated [61, 62], and the main agents used are mannitol and hypertonic saline (HTS). The supposed mechanism of action is to improve blood rheology and cerebral microvascular flow and create an osmolar gradient across the blood–brain barrier (BBB), favoring cerebral edema reabsorption. Mannitol is an osmotic diuretic, and at doses of 0.25 to 1 g/kg, it has been associated with effective ICP reduction in patients with brain injury, although there is little evidence of its use specifically in SAH [63]. HTS has a minimal diuretic effect, unlike mannitol. It can expand the intravascular volume and increase blood pressure and serum sodium levels. The HTS concentrations reported were between 3 and 23.5%. Equimolar doses decrease ICP similarly. The upper safety limit is often reported as either a serum sodium level of 155–160 mEq/L or a serum osmolality of 320 mOsm. According to guidelines for treating cerebral edema in SAH, a symptom-based bolus dosing is suggested rather than a sodium-target-based dosing [61].

In a retrospective cohort analysis of 68 patients with multiple intracranial pathologies, Koenig et al. reported that in 75% of cases, 30–60 mL of 23.4% NaCl was sufficient to reverse transtentorial herniation, and this was associated with a > 5% mEq/L rise in the serum sodium concentration or an absolute serum sodium level of > 145 mEq/L within 1 h after HTS administration [64]. A recent review [65] on HTS confirmed its efficacy in reducing refractory ICP in patients with SAH, although no recommendation on the dose, volume, and concentration was made. In addition, Bentsen et al. [66] found osmotherapy with HTS to attenuate both static ICP and ICP wave amplitude; of note, they report that in the majority of HTS infusions, the ICP wave amplitude target was not reached even though ICP and CPP targets were, a finding consistent with an unfavorable intracranial compliance state despite normal static values.

### Hypothermia and Barbiturate Coma

Hypothermia and barbiturate coma are tier 3, the last resource for refractory HICP, and are supported by anecdotal evidence at most. Moreover, in the Intraoperative Hypothermia for Aneurysm Surgery Trial, which included 1001 patients, hypothermia during aneurysm surgery, used as a prophylactic neuroprotective strategy, did not improve outcomes [67].

Fever is common after SAH [68–71] and is an independent predictor of poor outcomes because high brain temperatures increase cerebral metabolic demands in an already suffering brain. Therefore, in the absence of evidence of the beneficial effects of hypothermia, strict normothermia should instead be targeted [72].

Barbiturate coma is considered the last resource for refractory ICP because of the high rate of adverse events, including prolonged sedation due to the long half-life, metabolic derangements, respiratory and immunologic suppression, and cardiovascular events [7]. However, evidence is lacking for routine administration in patients with SAH with refractory HICP.

### Decompressive Craniectomy

Decompressive craniectomy is a critical procedure utilized to address elevated ICP in cases of ischemic stroke and TBI. Numerous studies have consistently demonstrated its effectiveness in reducing ICP and improving cerebral perfusion pressure (CPP). However, it is important to note that this procedure comes with the trade-off of a high likelihood of a poor functional outcome [73].

Currently, there is insufficient evidence to establish the superiority of decompressive craniectomy over medical management for treating severe refractory HICP in patients with SAH. Nevertheless, bilateral decompression remains a viable option as a rescue therapy when maximal medical management has been exhausted. It represents the most promising intervention in situations where clinicians have explored all other available alternatives and there is a diffuse cerebral pathology [74].

A preplanned subanalysis of the Synapse-ICU study recently demonstrated that aggressive strategies could reduce 6-month mortality in selected patients, but this effect was not evident in neurological outcomes [75].

## Practice Guidance

**Fig. 1:**
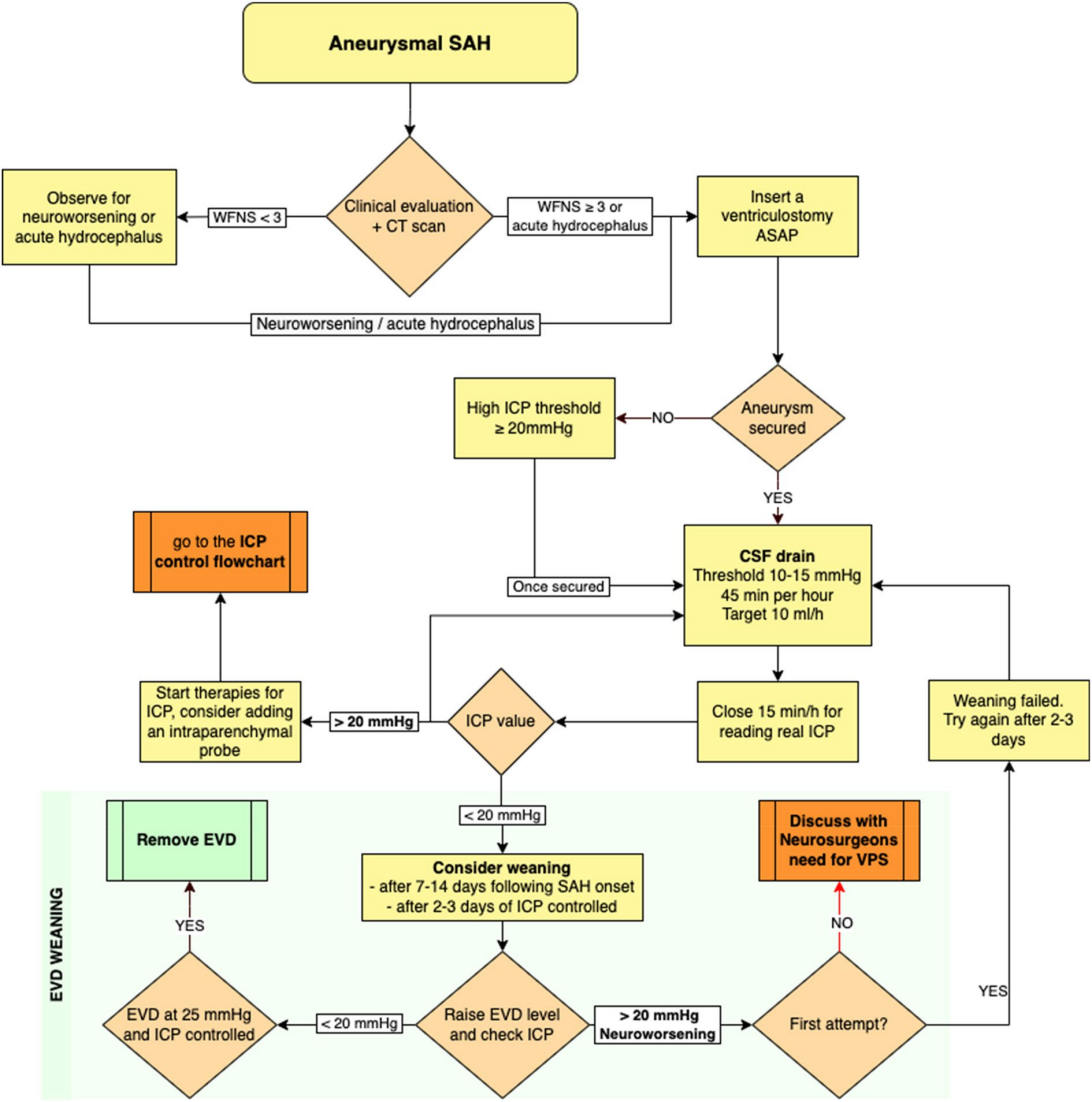
Flowchart summarizing the management of intracranial pressure (ICP) monitoring after aneurysmal subarachnoid hemorrhage (SAH). Details in the text

**Fig. 2:**
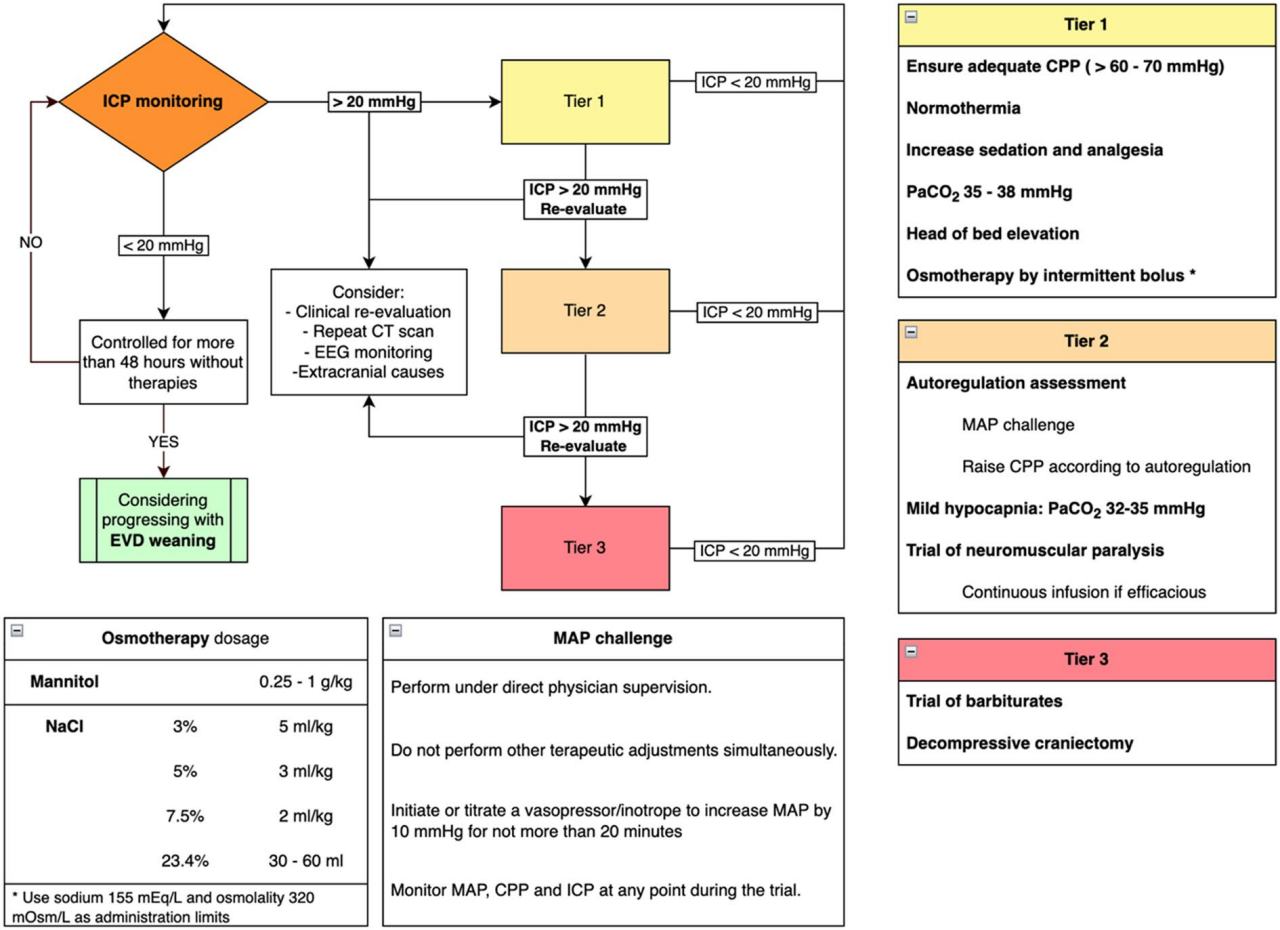
Strategies for controlling intracranial pressure (ICP). See text for details

Based on our clinical experience (summarized in Figs. 1 and 2), we recommend the following ICP management approach for patients with SAH:

- Insert an EVD before securing the aneurysm in patients with a World Federation of Neurological Surgeons score of ≥ 3 or acute hydrocephalus. In our unit, we use a combined EVD and intraparenchymal device.
- After EVD placement, set the drainage to high thresholds (> 20 mm Hg) or ICP until aneurysm obliteration to prevent aneurysmal transmural pressure reduction and rebleeding.
- After securing the aneurysm, use a continuous EVD drainage approach, leaving the drain open against a gradient of 10–15 mm Hg. Early blood clearance after aneurysmal SAH has been associated with less DCI [76]. Close the EVD for proper recording of ICP for 10–15 min every hour. Monitor the drained volume hourly and aim for a target of around 10 mL/hour. If a parenchymal device is available, record ICP continuously.
- If ICP is not controlled with CSF diversion alone, escalate to ICP management strategies following a stepwise approach. Rule out extracranial confounders, such as fever, high CO_2_ levels, venous drainage disturbances, or low CPP causing vasodilation, before escalating to the following:
- Increasing analgesia and sedation.
- Mechanical ventilation aiming for a PaCO_2_ of 35–38 mm Hg.
- Osmotic therapy (HTS or mannitol) administered in boluses.
- Further progression would include the following:
- After ensuring adequate analgosedation, perform a bolus trial of neuromuscular paralysis to check for benefits then progress to continuous infusion if such efficacy can be demonstrated.
- Autoregulation test with a mean arterial pressure challenge as described by the Seattle International Severe Traumatic Brain Injury Consensus Conference (SIBICC) consensus [77].
- Hyperventilation to a PaCO_2_ of 32–35 mm Hg.
- In case of persistent refractory intracranial hypertension:
- Tier 3 therapies involve testing the response to a bolus of barbiturates as a temporary measure to buy time, while engaging in discussions with the family about the potential benefits of decompressive craniectomy. Ideally, the decision to perform a decompressive craniectomy should be made in patients who are salvageable and have previously exhibited good clinical conditions.

At each level of therapeutic intensification, reevaluate the patient clinically and radiologically. Consider checking for epileptiform activity or nonconvulsive seizures requiring specific pharmacological treatment. When ICP is under control (usually after days 7–14), gradually wean from the EVD by raising the drain over several days until it reaches 25 mm Hg. Then, clamp and discontinue the EVD. This approach allows for a progressive rebalancing between CSF production and reabsorption pathways over 2–3 days. If this strategy fails, try the progressive closure again after a couple of days and discuss with neurosurgeons the need for a ventriculoperitoneal shunt [57].

## Future Directions

Our current understanding of ICP after SAH is limited, which poses a challenge to the effective management of this condition. Although ICP monitoring and management are crucial for patients with SAH, the scarcity of high-quality evidence to guide clinical decision-making is a significant obstacle. Future clinical trials should prioritize identifying optimal ICP management strategies and assessing their impact on patient outcomes.

Currently, patients with SAH are managed using stepwise approaches to ICP management. However, technological advances and multimodal monitoring may enable personalized ICP management based on individual patient characteristics and measured parameters, such as brain electrical activity and tissue oxygenation.

Artificial intelligence is a promising tool for the early prediction of intracranial hypertension and for guiding personalized management. Artificial intelligence algorithms can analyze patient data, such as vital signs, laboratory values, and imaging, to predict the likelihood of developing intracranial hypertension and suggest appropriate interventions.

Furthermore, there is growing interest in developing noninvasive methods for ICP monitoring because of the risks associated with invasive monitoring devices, such as infection, hemorrhage, and other complications. Although some noninvasive methods, such as transcranial Doppler ultrasound, ONSD measurement, and magnetic resonance imaging–based methods, have shown disappointing results, continued research and development may lead to more effective techniques.

It is important to consider other factors contributing to poor outcomes in patients with SAH, such as cerebral edema and inflammation. Combination therapy targeting multiple pathways simultaneously may be more effective in improving outcomes for these patients. Therefore, managing intracranial hypertension in patients with SAH should involve a holistic approach that addresses multiple factors contributing to poor outcomes.

## Conclusions

Intracranial hypertension significantly contributes to poor outcomes in patients with aneurysmal SAH. However, the indications for ICP monitoring and management in aneurysmal SAH are highly variable and lack clear guidelines. ICP monitoring is crucial in managing patients with aneurysmal SAH, including those with good grades. Invasive monitoring devices are the gold standard, and in cases of hydrocephalus, an EVD for CSF diversion is necessary, along with the possible use of an intraparenchymal probe to allow continuous ICP monitoring. Although specific studies are lacking, using a stepwise approach, ICP-guided and CPP-guided management can be derived from observations in other pathological entities. Therefore, further research in aneurysmal SAH is necessary to define the best management strategies for ICP in these patients.
